# Time–Frequency-Analysis-Based Blind Modulation Classification for Multiple-Antenna Systems

**DOI:** 10.3390/s21010231

**Published:** 2021-01-01

**Authors:** Weiheng Jiang, Xiaogang Wu, Yimou Wang, Bolin Chen, Wenjiang Feng, Yi Jin

**Affiliations:** 1 School of Microelectronics and Communication Engineering, Chongqing University, Chongqing 400044, China; xiaogangwu@cqu.edu.cn (X.W.); chenbolin.cqu@gmail.com (B.C.); fengwj@cqu.edu.cn (W.F.); 2 Armour College of Engineering, Illinois Institute of Technology, Chicago, IL 60616, USA; ywang461@hawk.iit.edu; 3 Xi’an Branch of China Academy of Space Technology, Xi’an 710100, China; john.0216@163.com

**Keywords:** time–frequency analysis, blind modulation classification, multiple-antenna systems, RGB spectrogram image

## Abstract

Blind modulation classification is an important step in implementing cognitive radio networks. The multiple-input multiple-output (MIMO) technique is widely used in military and civil communication systems. Due to the lack of prior information about channel parameters and the overlapping of signals in MIMO systems, the traditional likelihood-based and feature-based approaches cannot be applied in these scenarios directly. Hence, in this paper, to resolve the problem of blind modulation classification in MIMO systems, the time–frequency analysis method based on the windowed short-time Fourier transform was used to analyze the time–frequency characteristics of time-domain modulated signals. Then, the extracted time–frequency characteristics are converted into red–green–blue (RGB) spectrogram images, and the convolutional neural network based on transfer learning was applied to classify the modulation types according to the RGB spectrogram images. Finally, a decision fusion module was used to fuse the classification results of all the receiving antennas. Through simulations, we analyzed the classification performance at different signal-to-noise ratios (SNRs); the results indicate that, for the single-input single-output (SISO) network, our proposed scheme can achieve 92.37% and 99.12% average classification accuracy at SNRs of −4 and 10 dB, respectively. For the MIMO network, our scheme achieves 80.42% and 87.92% average classification accuracy at −4 and 10 dB, respectively. The proposed method greatly improves the accuracy of modulation classification in MIMO networks.

## 1. Introduction

The increase in communication demands and the shortage of spectrum resources have caused the cognitive radio (CR) and multiple-input multiple-output (MIMO) techniques to be implemented in wireless communication systems. As one of the essential steps of CR, modulation classification (MC) is widely applied in both civil and military applications, such as spectrum surveillance, electronic surveillance, electronic warfare, and network control and management [1]. It improves radio spectrum utilization and enables intelligent decision-making for context-aware autonomous wireless spectrum monitoring systems [2]. However, most of the existing MC methods are focused on single-input single-output (SISO) scenarios, which cannot be directly applied when multiple transmitting antennas are equipped at the transceivers [3]. Therefore, it is crucial to research the performance of the MC method for MIMO communication systems.

Traditional MC approaches for the SISO systems discussed in the literature can be classified into two main categories: likelihood-based (LB) approaches and feature-based (FB) approaches [4]. The LB approaches can theoretically achieve optimal performance, as they compute the likelihood functions of the different modulated signals to maximize the classification accuracy. However, they have a very high computational complexity and require prior information, such as the channel coefficient [5,6]. Hence, the LB approaches cannot be directly applied in fast modulation classification and blind modulation classification (BMC). By contrast, the FB approaches cannot obtain the optimal result, but they have lower computational complexity and do not require prior information [7]. The FB methods usually include two steps: feature extraction and classifier design. The higher-order statistics, instantaneous statistics, and other features are calculated in the feature extraction. Then, the popular classification methods, such as decision trees [8], support vector machine [9,10], and artificial neural networks (ANNs) [11,12], are adopted as the classifiers. With the rapid rise of artificial intelligence and the emerging requirements of intelligent wireless communication, deep-learning-based approaches are now becoming widely studied and used in different aspects of wireless communication, such as the transceiver design at the physical layer [13] and BMC problems [7,14,15,16,17,18]. More specifically, Rajendran et al. [15] proposed a new data-driven model for BMC based on long short-term memory (LSTM), which learned the features from the time-domain amplitude and phase information of the modulation schemes and yielded an average classification accuracy close to 90% for signal-to-noise ratios (SNRs) from 0 to 20 dB. In addition, the time–frequency features have also been applied to the deep-learning-based BMC problem. In [7], the Resnet model was adopted as the classifier, and the authors presented an approach for fusing the red–green–blue (RGB) spectrogram images and the handcrafted features of the modulated signals to obtain more discriminating features. The experimental results demonstrated that the proposed scheme has a superior performance. The latest research indicates that deep-learning-based MC methods achieve higher accuracy than the traditional LB and FB approaches for SISO systems [7,19].

Though right now, multiple antennas have been adopted by many mobile communication systems, our investigation and survey indicate that the MC for MIMO systems is less discussed than for the SISO system. Recent studies about this topic are summarized in Table 1, and they are characterized and distinguished by the scenario, method, features, modulation classes, SNR, and accuracy. From this table, we can note that most of the proposed approaches are FB-based; concretely, the authors in [20,21] proposed similar methods for the MC of MIMO transceiver systems that calculate the higher-order statistical moments and cumulants of the received signals. Then, an artificial neural network is employed to classify the modulation types. In [22], a clustering classifier based on centroid reconstruction is presented to identify the modulation scheme with an unknown channel matrix and noise variance in MIMO systems. The simulation results showed that their algorithm could obtain excellent performance, even at low SNRs and with a very short observation interval. To deal with the BMC problem and the two major constraints in the railway transmission environment (i.e., the high speeds and impulsive nature of the noise), Kharbech et al. [23] proposed a feature-based process of blind identification that includes three parts: impulsive noise mitigation, feature extraction, and classification. By analyzing the correlation functions of the received signals for certain modulation formats, Mohamed et al. resolved the BMC problem in single- and multiple-antenna systems operating over frequency-selective channels in [24] and the BMC problem in the Alamouti space-time block code (STBC) System [25].

More interesting studies about this topic concern the modulation classification for MIMO orthogonal frequency division multiplexing (OFDM) systems, as the MIMO OFDM has been widely adopted by many commercial standards, such as LTE and the next Wifi. For this problem, different approaches, such as the approximate Bayesian inference method, the Gibbs sampling-based method, and the joint independent component analysis (ICA) with support vector machines (SVMs) method, were proposed by Liu et al. in [26,27,28,29] for the MIMO OFDM signals. However, all these studies are traditional feature-based or likelihood-based approaches.

In fact, from the aforementioned MIMO-based systems, we note that it is difficult to directly apply deep learning to the raw in-phase and quadrature (IQ) data or the time-domain amplitude and phase data, since the overlapped signals at the receiver of the MIMO system destroy the statistical features [30]. Hence, it is crucial to extract the distinguishable features or convert the raw signals for BMC in MIMO systems. The time–frequency analysis methods can jointly analyze the time-domain and frequency-domain features of signals, and the different modulation types have distinct time-domain and frequency-domain features. Hence, in this paper, in order to overcome the effect of the overlapping signals at the receiver, we analyze the time–frequency features of the modulated signals to resolve the BMC problem in MIMO systems. First, the time–frequency analysis method based on the windowed short-time Fourier transform (STFT) [31] is employed to generate the spectrum of the MIMO-modulated signals. Then, the spectrum with different time windows is converted to a grayscale image, and this grayscale image is further transferred to the RGB spectrogram image [32]. Second, a fine-tuned AlexNet-based convolutional neural network (CNN) model is introduced to learn the features from the RGB spectrogram images. The modulation scheme of each receiving stream among the receiving MIMO signals is identified in this stage. Finally, the previously produced decisions are merged to form the final result. In addition, this method can be simplified to directly apply to SISO systems. The simulation results show that the proposed method achieves a superior performance in low-SNR scenarios for both the MIMO system and the SISO system. Particularly, the proposed method obtains −80.42% accuracy at SNR = −4 dB for the MIMO network, which is the highest accuracy compared with the existing works, as mentioned in Table 1.

This paper is organized as follows. The signal models of the MIMO and SISO systems and the STFT-based time–frequency analysis method are introduced in Section 2. Section 3 presents the BMC scheme for the MIMO systems, including the proposed CNN model and the decision method. Then, the RGB spectrogram image and the classification performance in different scenarios are analyzed in Section 4. Finally, conclusions are drawn in Section 5.

## 2. Signal Model and Time–Frequency Analysis Method

In this section, we define the MIMO signal model, and then the simplified SISO signal model is derived. Then, the STFT-based time–frequency analysis method is introduced to generate the spectrogram image of the MIMO modulated signals.

### 2.1. MIMO Signal Model

We consider a MIMO-based single-carrier wireless communication system with $N_{t}$ transmitting antennas and $N_{r}$ receiving antennas. For simplification, herein, we only consider the flat-fading and time-invariant MIMO channel (though in the performance analysis section, we will extend the discussion to the frequency-selective and the time-varying channel models) (It is important to point out that this does not mean that our proposed method can be directly extended to the MIMO OFDM system. In fact, how one can extend the proposed method to the MIMO OFDM system without significant increasing the complexity of the system, i.e., the information feedback from the receiver to the transmitter, is an interesting issue and is worth further study). Therefore, the MIMO channel $H\in C^{N_{r}\times N_{t}}$ is defined as

(1) $$H=\left[\begin{array}{ccc} h_{11} & \cdots & h_{1N_{t}}\\ \vdots & \ddots & \vdots \\ h_{N_{r}1} & \cdots & h_{N_{r}N_{t}} \end{array}\right],$$

where $h_{ij}$ represents the channel coefficient between the *j*-th transmitting antenna and the *i*-th receiving antenna. The channel matrix H is assumed to be full-column rank. Then, the received signals can be further represented as

(2) y=Hx+n,

where $x=[x_{1}\left(t\right),\ldots ,$$x_{j}\left(t\right),\ldots ,X_{N_{t}}{\left(t\right)]}^{T}$ is $N_{t}\times 1$ transmitted data, and $x_{j}\left(t\right)$ represents the transmitted modulated signal at the *j*-th transmitting antenna. Likewise, $y={\left[y_{1}\left(t\right),\ldots ,y_{i}\left(t\right),\ldots ,y_{N_{r}}\left(t\right)\right]}^{T}$ represents the $N_{r}\times 1$ received data vector, $y_{i}\left(t\right)$ is the received signal at the *i*-th receiving antenna, and $n={\left[n_{1}\left(t\right),\ldots ,n_{i}\left(t\right),\ldots ,n_{N_{r}}\left(t\right)\right]}^{T}$ represents the additive white Gaussian noise (AWGN) vector; each element $n_{i}\left(t\right)$ of n is an identically and independently distributed (i.i.d.) random variable with zero mean and variance $\sigma^{2}$ (i.e., $n_{i}\left(t\right)∼N\left(0,\sigma^{2}\right)$) [20]. In order to obtain the RGB spectrogram image of $y_{i}\left(t\right)$, the datasets generated in this paper are time-domain signals [7], instead of the baseband signals used in [33,34].

In this paper, the considered original digital modulated signals x(t) may be the multiple amplitude-shift keying (MASK), multiple frequency-shift keying (MFSK), multiple phase-shift keying (MPSK), and quadrature amplitude modulation (QAM) signals [35]. The time-domain expressions of all these modulated signals are presented in Appendix A.1.

### 2.2. SISO Signal Model

When $N_{r}=N_{t}=1$, the MIMO-based signal model in Section 2.1 can be converted into an SISO-based signal model. The received signals corrupted by the AWGN in the SISO system can then be represented as

(3) y(t)=hx(t)+n(t),

where x(t) represents the original digital modulated signals, y(t) represents the digital modulated signals over the wireless channel, *h* represents the channel attenuation coefficient, and n(t) denotes the AWGN.

### 2.3. STFT-Based Time–Frequency Analysis

In this paper, the STFT is adopted in the modulated signal analysis. That is, we use STFT to analyze the frequency and phase of local sections of the time-varying modulated signals with a time window function [36]. Then, the spectrogram image (the visual representation of the frequency spectrum of a signal) is constructed. In this subsection, we introduce the theory of the STFT, and then we present the method to generate the STFT-based RGB spectrogram image for the modulated signals.

#### Generating the STFT-Based RGB Spectrogram Image for the Modulated Signals

**Figure 1:**
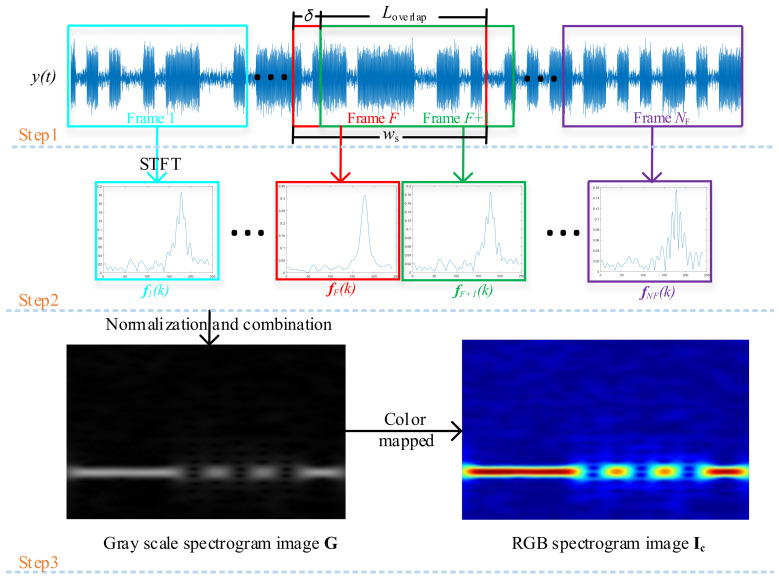
The flow chart of the short-time Fourier transform (STFT)-based time–frequency analysis.

In order to perform the STFT (the details of this transformation are shown in Appendix A.2) and obtain the spectrogram image of the modulated signals, we follow the process illustrated in Figure 1. That is, we divide a given discrete modulated signal vector y(n) of length *L* into highly overlapped frames, each with length $w_{s}$, and then use the windowed STFT to obtain the spectral vector f, where y(n) is obtained by sampling the received modulated signal y(t). Hence, the signal in the current frame, $y_{F}\left(n\right)$, is

(4) $$y_{F}\left(n\right)=y\left(F\delta +n\right)w\left(n\right),n=0,\ldots ,w_{s}-1,$$

where *F* is the current frame and w(n) is the window function. The window function can be hamming, hanning, or blackman, and we choose hamming in this paper [37]. Then, the δ is the incrementation between two consecutive frames, which is calculated by

(5) $$\delta =w_{s}-L_{overlap}.$$

Herein, $L_{overlap}$ ($L_{overlap}<w_{s}<L$) is the length of overlapping signals between two consecutive frames, and the number of frames $N_{F}$ can be calculated by

(6) $$N_{F}=\frac{L-L_{overlap}}{\delta }=\frac{L-L_{overlap}}{w_{s}-L_{overlap}}.$$

The larger the $L_{overlap}$, the greater the $N_{F}$, and hence, the higher the time resolution of the STFT.

The hamming window function w(n) is defined as

(7) $$w\left(n\right)=\left[0.54-0.46cos\left(\frac{2\pi n}{w_{s}-1}\right)\right]R_{w_{s}}\left(n\right),$$

where $R_{w_{s}}\left(n\right)$ is a rectangular window with length $w_{s}$.

Based on (4), we can obtain the spectral magnitude vector $f_{F}$ of the current frame *F*,

(8) $$f_{F}\left(k\right)=\sum_{n=0}^{w_{s}-1}y_{F}\left(n\right)e^{-j2\pi nk/N},k=1,\ldots ,\left(N/2-1\right)$$

where N/2−1 is the number of points of the Fourier transform. The larger the *N*, the higher the frequency resolution of the STFT. Therefore, the linear value of the spectral magnitude vector is obtained as

(9) $$S\left(k,F\right)=|f_{F}\left(k\right)|.$$

The linear value of the spectral magnitude vector can be normalized in the range of [0, 1] as

(10) $$G\left(k,F\right)=\frac{S(k,F)-min(S)}{max(S)-min(S)}.$$

By combining the normalized linear spectral magnitude vector G(k,F) of all the frames as

(11) $$G=[G{\left(k,1\right)}^{T};\ldots ;G{\left(k,F\right)}^{T};\ldots ;G{\left(k,N_{F}\right)}^{T}],$$

We can obtain the time–frequency matrix $G\in C^{\left(N/2-1\right)\times N_{F}}$. This matrix is a grayscale image of the spectral magnitude vector; the size of this image is $\left(N/2-1\right)\times N_{F}$, the horizontal axis of this image represents time, and the vertical axis represents frequency.

Next, the grayscale image is quantized into its RGB components; the mapping type is the jet in MATLAB r2016b [38]. The mapping is expressed as

(12) $$I_{c}=f_{map}\left(G\right),$$

where $I_{c}$ is the RGB spectrogram image and $f_{map}$ is the non-linear jet quantization function [32]. It is worth noting that, to facilitate the observation and analysis of the RGB spectrogram image, we deploy the color mapping in this paper. This step can be omitted in practical applications.

For the STFT, by adjusting the values of the window length $w_{s}$ and overlapping signal length $L_{overlap}$, we can tune the time resolution of the RGB spectrogram image. Moreover, by adjusting the number of points of the Fourier transform N/2−1, we can also tune the frequency resolution of the RGB spectrogram image.

## 3. Proposed BMC Scheme

**Figure 2:**
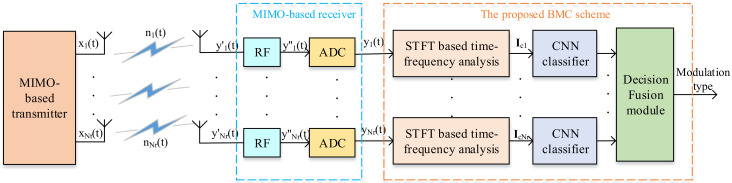
Block diagram of the proposed MIMO modulation classification scheme.

In this section, a time–frequency analysis is conducted and a deep-learning-based BMC scheme is proposed. The block diagram of the proposed BMC scheme is shown in Figure 2, which shows four modules: signal generator, time–frequency analysis, CNN classifier, and decision fusion. The signal generator outputs the modulated signals $x_{i}\left(t\right)$ (with the same modulation type) for each transmitting antenna [20]. This process was described in Section 2.1 and Section 2.2. Then, the time–frequency analysis is performed for the received signal $y_{i}\left(t\right)$ for each receiving antenna, which generates the RGB spectrogram image $I_{ci}$ (partially described in Section 2.3). Next, the AlexNet-based CNN classifier is trained based on a number of RGB spectrogram images in the training stage, and the modulation type of each received signal $y_{i}\left(t\right)$ is identified in the test stage. Finally, the decisions of different signal branches are combined by the decision fusion module for the final decision. In the next three sections, we will illustrate in detail the procedures of the time–frequency analysis, CNN-based classifier, and decision fusion.

### 3.1. Time–Frequency Analysis for Received Signals

The flow chart of STFT-based time–frequency analysis is shown in Figure 1. First, using the amplitude-shift keying (ASK) signal as an example, the received signal y(t) is divided into $N_{F}$ frames by the hamming window w(n) with length $w_{s}$, the details of which are described in Equations (4)–(7). Second, the spectrum of the windowed signal is obtained by its Fourier transform. Third, by normalizing and combining the linear spectral magnitude vector, the grayscale spectrogram image G is obtained (the size of the related grayscale matrix is $\left(N/2-1\right)\times N_{F}$). Finally, to accommodate the input layer of AlexNet and improve the distinguishability of the spectrogram image, the grayscale spectrogram image is mapped onto the RGB spectrogram image $I_{c}$ (the size of the related RGB matrix is $\left(N/2-1\right)\times N_{F}\times 3$). Then, the RGB matrix is cut or padded into 227×227×3 before being fed into the CNN.

### 3.2. AlexNet-Based CNN Classifier

**Figure 3:**
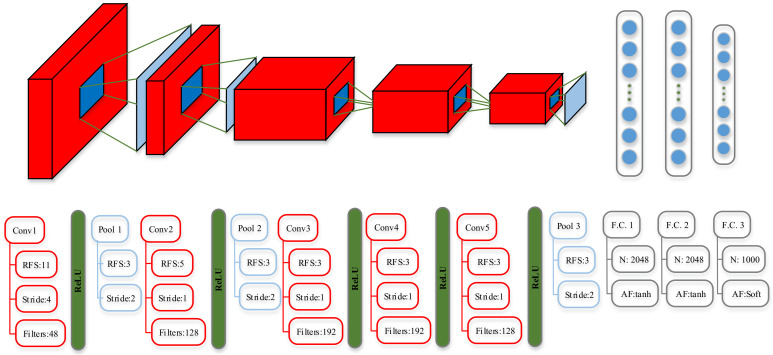
Architecture of AlexNet (Conv: convolution layer, Pool: pooling layer, F.C.: fully connected layer, RFS: receptive field size, N: number of neurons in fully connected layer, AF: activation function, Soft: softmax) [40].

In our proposed BMC scheme, AlexNet, which is utilized for object detection [39] and was the winner of the 2012 ImageNet Large-Scale Visual Recognition Challenge (ILSVRC), is adopted as the classifier. The network architecture of AlexNet is shown in Figure 3 [40].

As depicted in Figure 3, AlexNet contains eight layers; the first five are convolutional and the remaining three are fully connected. The output of the last fully connected layer is fed to a 1000-way softmax that produces a distribution over the 1000 class labels [39]. AlexNet uses the rectified linear unit (ReLU) as the activation function of the CNN. In practice, the dropout and max pooling techniques are applied to the CNN. AlexNet has an excellent performance in visual tracking and object detection due to its capability in sensing the pattern position on the image. Therefore, considering that the spectrogram image has rich pattern position information, it is sensible to choose AlexNet as the classifier network.

The motivation for transfer learning comes from the fact that people can intelligently apply knowledge learned previously to solve new problems faster or with better solutions [41]. In order to utilize the pretrained AlexNet, transfer learning is employed to fine-tune AlexNet and accelerate the training process. The last layer of the pretrained AlexNet network in Figure 3 is configured with 1000 classes, and this layer must be fine-tuned to accommodate the new classification task. First, all layers except the last layer are extracted; then, the last layer is replaced with a new fully connected layer that contains eight neurons (i.e., the number of modulation categories in this paper). In the end, the parameters of the activation layer and the classification output layer are set to accommodate the new classification task. Therefore, with such fine-tuning, the output of AlexNet can precisely perform the modulation classification of the received signals. The training hyperparameters are listed in Table 2; concretely, the minimum of the batch size is set to 10, the maximum of the training epoch is set to 10, and the learning rate is set to $1.0\times 10^{-4}$, respectively.

### 3.3. Decision Fusion

Since there are multiple antennas at the receiver of the MIMO network, it is possible for each branch to cooperate with the others to achieve higher identification reliability [20]. As shown in Figure 2, the $N_{r}$ received signals are classified independently because the influences of signal overlapping, interchannel noise, and random phase shifting may cause each received signal to be identified as a different modulation type. This may lead to incorrect identification results. The decision fusion among all the receiving antennas aims to improve the average classification accuracy. The decision vector of the *i*-th received signal, $d_{i}$, can be defined as

(13) $$d_{i}=\left[d_{i1},\ldots ,d_{ik},\ldots ,d_{iK}\right],$$

where *K* is the number of modulation types, $d_{ik}$ is the probability of identifying the received signal $y_{i}\left(t\right)$ as modulation type *k*, and $d_{ik}$ meets the following condition:

(14) $$\sum_{k=1}^{K}d_{ik}=1.$$

Therefore, the modulation type $m_{i}$ of the received signal $y_{i}\left(t\right)$ is the modulation type that has the maximum probability. The modulation type with maximum probability can be defined as a set M as follows:

(15) $$M=\underset{k\in \{1,\ldots ,K\}}{argmax}d_{ik}.$$

Note that there are two cases for the above equations: (1) The maximum probability is unique, i.e., |M|=1, and the modulation type of the *i*-th received signal is the element of M; (2) the maximum probability is not unique, i.e., |M|≥2, and the modulation type of the *i*-th received signal is randomly chosen from M.

Hence, the decision fusion can be converted into the problem of deciding the final modulation type *m* according to $m_{i}$, $i=1,2,\ldots ,N_{r}$. The fusion rule at the fusion module can be OR, AND, or majority rule, which can be generalized as the “n-out-of-$N_{r}$ rule” [42]. That is, a certain modulation scheme is identified when a classifier is decided on among the $N_{r}$ classifiers. Take the $N_{r}=4$ as an example; the possible modulation types form the set M= {2PSK, 4PSK, 8PSK}. If there are more than three classifiers, the modulation type is identified as 2PSK (4PSK or 8PSK); then, the final modulation type is 2PSK (4PSK or 8PSK). If there are two classifiers, the modulation type is identified as 2PSK, and the modulation types of the other two classifiers are identified as 4PSK and 8PSK, respectively; then the final decision is 2PSK. In addition, if the two classifiers identify the modulation type as 2PSK and the other two classifiers identify the modulation type as 4PSK (or 8PSK), the decision fusion center will randomly choose a modulation type between 2PSK and 4PSK (or 8PSK) as the final result.

## 4. Performance Analysis

In this section, the proposed time–frequency analysis and deep-learning-based BMC algorithm are tested under different modulation schemes for both the SISO and MIMO scenarios. Specifically, the channel coefficient *h* randomly takes values from [0,1] over multiple symbols, and the AWGNs with different SNRs are added into the modulated signals for both the SISO and MIMO scenarios. For the MIMO system, random phase shifts within one symbol interval are considered for the MIMO scenario. Without any other statements, the MIMO antenna configurations are $N_{t}=2$ and $N_{r}=4$. In addition, the 2ASK, 2FSK, 2PSK, 4ASK, 4FSK, 4PSK, 8PSK, and 16QAM modulation schemes are considered, unless otherwise stated. The parameters of the modulated signals are assigned as follows. The sampling frequency $f_{s}$ is 16 KHz, the carrier frequency $f_{c}$ is 2 KHz, the symbol rate $f_{b}$ is 100 Hz, and the length of the original digital signal is 14 (i.e., each modulated signal contains (16,000/100) × 14 = 2240 sample points). In addition, in the training stage, 100 modulated signals for each modulation type and SNR are randomly generated for both the SISO and MIMO scenarios, in which the SNR varies from −4 to 10 dB at intervals of 2 dB [7]. In the test stage, 100 modulated signals for each modulation type and SNR are randomly generated. All the signal samples were generated in MATLAB 2017b, and the training and testing of AlexNet are based on the MATLAB neural network toolbox. Additionally, the parameters to generate the RGB spectrogram image were set as $w_{s}=320$, $L_{overlap}=315$, δ=5, and N=2048.

We now discuss how the modulation order, SNR, and overlapping of the MIMO signals influence the RGB spectrogram image of the modulated signals. Then, the classification performance of the proposed scheme is validated for different scenarios.

### 4.1. RGB Spectrogram Image of the Modulated Signals

**Figure 4:**
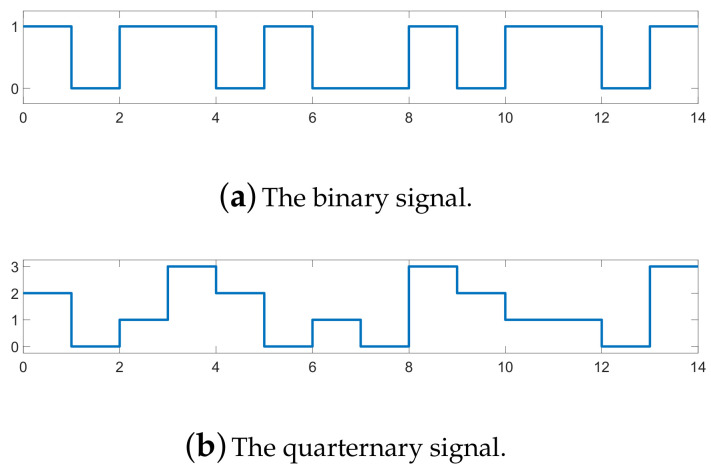
Digital signal sequence before modulation.

In this subsection, in order to simplify the analysis, we select only certain binary and quaternary digital signal sequences (as shown in Figure 4) to generate the RGB spectrogram image. The binary signal Figure 4a is used to generate the two-order modulated signals (i.e., 2ASK, 2FSK, and 2PSK), and the quaternary signal Figure 4b is used for the four-order modulated signals (i.e., 4ASK, 4FSK, and 4PSK).

#### 4.1.1. RGB Spectrogram Image of the Modulated Signals with Different Modulation Orders

**Figure 5:**
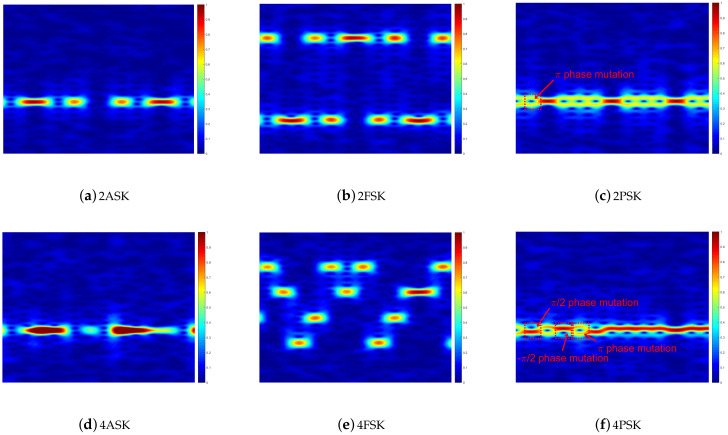
Red–green–blue (RGB) spectrogram images of modulated signals in single-input single-output (SISO) networks at a signal-to-noise ratio (SNR) of 10 dB.

We first evaluate how the modulation order affects the RGB spectrogram image at an SNR of 10 dB for the SISO scenario. The considered modulation schemes are ASK, FSK, and PSK, which are shown in Figure 5. They are analyzed separately as follows.

First of all, the RGB spectrogram image is a time–frequency distribution image of the modulated signal. The horizontal axis of this image represents time and the vertical axis represents frequency. In addition, the color of the RGB spectrogram image represents the value of the normalized spectral magnitude (i.e., the values corresponding to blue and red are zero and one, respectively).

Figure 5a,b show the RGB spectrogram image of the ASK-modulated signals. The power of the ASK-modulated signals concentrates on one frequency band in the image, and the power in the image is discontinuous over time. In addition, the color in the image is blue when the digital signal sequence is at the zero level in Figure 4, and it is red when the digital signal sequence is at a non-zero level, which corresponds to the values of the spectral magnitude. In addition, compared with the 2ASK signal, the spectral magnitude of the 4ASK signal has a larger average value (i.e., more pixels in the 4ASK RGB spectrogram image have a value of 1).

Figure 5b,e show the RGB spectrogram image of the FSK-modulated signals at an SNR of 10 dB. The spectral magnitude of the 2FSK-modulated signals has a larger value over two sub-bands, and the spectral magnitude of the 4FSK-modulated signals has a larger value over four sub-bands. For the FSK signals, the modulation order is equal to the number of modulated frequencies, which is the number of sub-bands in the RGB spectrogram image.

The RGB spectrogram images of the PSK-modulated signals are shown in Figure 5c,f. The phase mutation of the modulated signals is captured in the RGB spectrogram images. Specifically, Figure 4a and Figure 5c both have the π-phase mutation in the 2PSK-modulated signal from 0 to 1 and from 1 to 0 in the binary digital signal sequences. The π-phase mutation decreases the value of the power spectral density at the modulated frequency, which appears as a “ring” in the RGB spectrogram image. Similarly, comparing Figure 4b and Figure 5f, the π/2- and 3π/2-phase mutations also partly decrease the value of the power spectral density at the modulated frequency, but they appear as a “half-ring” in the RGB spectrogram image. Therefore, modulated signals with different modulation orders have different time–frequency features, and it is reasonable to classify the modulated signals using the time–frequency analysis.

#### 4.1.2. RGB Spectrogram Image of the Modulated Signals for the MIMO Channels

**Figure 6:**
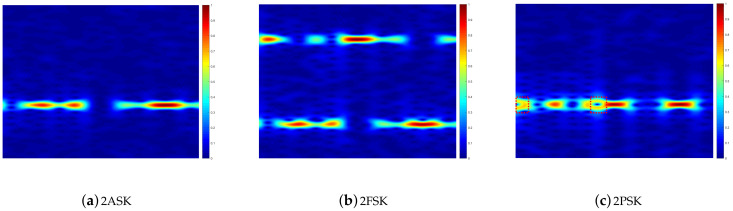
RGB spectrogram images of the modulated signals in MIMO networks at SNR = 10 dB.

We now analyze how the MIMO channel influences the RGB spectrogram image of the modulated signals. The 2ASK, 2FSK, and 2PSK modulation schemes are discussed herein. The antenna configuration for the MIMO system is $N_{t}=2$ and $N_{r}=4$; then, the random channel attenuation assigns a value from [0,1], random phase shifts within one symbol interval are considered for the MIMO scenario, and the AWGNs with 10 dB SNRs are added into the modulated signals. In addition, a multiplexing-based transmission scheme is adopted for the MIMO system. Specifically, two transmitting antennas send two independent data streams, but with the same modulation scheme (e.g., 2ASK, 2FSK, or 2PSK). The result is shown in Figure 6.

A comparison of Figure 5 and Figure 6 shows that, for all the modulated signals, the signal overlapping of the MIMO system has no effect on the power distribution of the modulated signals in the frequency domain, but the power distribution over the time domain is changed. The latter can be explained by the fact that the overlapping of different transmitted signals partly destroys the time–frequency characteristics of raw modulated signals. In spite of this, some crucial time–frequency characteristics are not destroyed by the MIMO signals overlapping, such as the “ring” that is caused by the phase mutation in the 2PSK signal (shown in Figure 5c and Figure 6c). Hence, the overlapping of modulated signals partially destroys the time–frequency characteristics, but some of the crucial time–frequency characteristics are still preserved in the RGB spectrogram image. Therefore, the RGB spectrogram image can still be used to identify the modulation type, even in the MIMO scenario.

#### 4.1.3. RGB Spectrogram Image of the Modulated Signals with Different SNRs for the MIMO Channels

**Figure 7:**
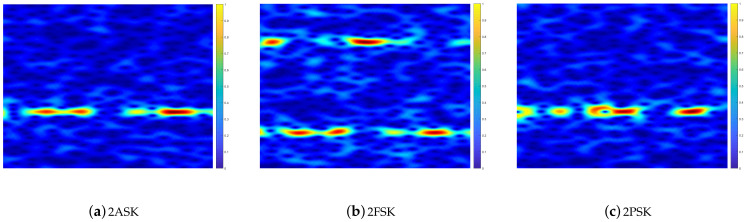
RGB spectrogram images of modulated signals in MIMO networks at SNR = −4 dB.

In this section, only the two-order modulation schemes are analyzed for different SNRs of the RGB spectrogram image for the MIMO network. For the 2ASK-modulated signals with SNR = 10 dB and SNR = −4 dB, the corresponding RGB spectrograms are shown in Figure 6a and Figure 7a, respectively. For the 2ASK-modulated signals, as the noise power increases, the components of the noise power become more prominent, as shown by the white patches in the RGB spectrogram image. However, the main features of the RGB spectrogram images of the 2ASK modulated signals are not destroyed. That is, the power distribution of the 2ASK-modulated signals is still concentrated in one sub-band in the RGB spectrogram image. In addition, the distribution of the power values of the power spectral density are almost the same at different SNRs. Similarly, the RGB spectrograms for the 2FSK- and 2PSK-modulated signals with SNR = 10 dB and SNR = −4 dB are shown in Figure 6b and Figure 7b and Figure 6c and Figure 7c, respectively. From these figures, we can conclude that increases in the noise power do not destroy the main features of the RGB spectrogram images of these modulated signals, and thus, they can be used as the features for modulation classification, even in the low-SNR region.

### 4.2. Classification Accuracy of the Proposed Scheme

The classification accuracy of the proposed scheme is tested and verified for both the SISO and MIMO scenarios. We first randomly generate the data stream, and then it is modulated and passed through the MIMO or SISO channels. In order to verify the performance of the proposed scheme, some benchmark schemes are introduced, such as the SqueezeNet-based method [43], the GoogleNet-based method [44], the scheme based on the smooth pseudo Wigner–Ville distribution (SPWVD) proposed in [7], and the scheme based on the Wigner–Ville distribution (WVD) proposed in [31].

#### 4.2.1. Classification Accuracy in the MIMO Scenario

**Figure 8:**
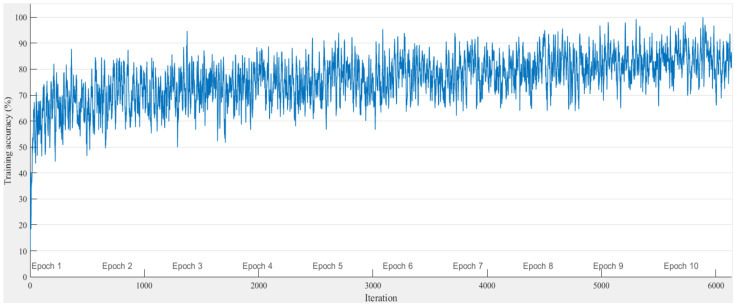
The training accuracy of network.

**Figure 9:**
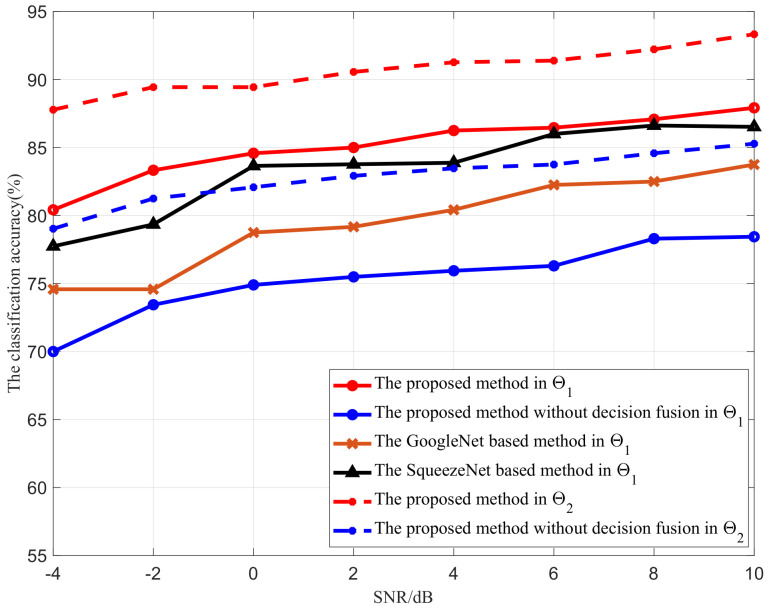
Classification accuracy versus SNR in MIMO networks.

The classification performance of the proposed scheme for the MIMO scenario is now verified. In order to better understand the performance of the proposed scheme, the model is trained and tested with two data sets, i.e., one for the modulation set $\Theta_{1}$ = {2ASK, 2FSK, 2PSK, 4ASK, 4FSK, 4PSK, 8PSK, 16QAM} and another for a smaller modulation set $\Theta_{2}$ = {2ASK, 2FSK, 2PSK, 4ASK, 4FSK, 4PSK}. In the training stage, Figure 8 shows the training accuracy versus the iterations in $\Theta_{1}$; as the number of iterations increases, the neural network converges gradually. In the testing stage, the SNR of the modulated signals is varied from SNR = −4 dB to SNR = 10 dB, and the result is shown in Figure 9. For both scenarios with and without the decision fusion module, the classification accuracy of the proposed scheme increases as the SNR of the modulated signals increases, which is consistent with the theoretical analysis. However, by introducing the decision fusion module, a 10% performance improvement in the classification accuracy can be achieved. In particular, the proposed scheme can achieve 80.42% and 87.92% accuracy at −4 and 10 dB SNR in $\Theta_{1}$, and 87.78% and 93.33% accuracy at −4 and 10 dB SNR in $\Theta_{2}$. In addition, the proposed scheme is also compared with the SqueezeNet- and GoogleNet-based schemes. The detailed hyperparameters are shown in Table 3. From this, we can note that the proposed scheme outperforms both the SqueezeNet- and GoogleNet-based methods. This can be explained as the fact that the decision fusion module and the excellent representational capability of AlexNet together enhance its classification accuracy. Moreover, we know that GoogleNet has the most training parameters and SqueezeNet has the least. In the training stage, since GoogleNet has the most training parameters and the training sets are not big enough, the complex GoogleNet can not fit well, and the generalization error of GoogleNet is higher than those of both SqueezeNet and AlexNet. SqueezeNet has the least training time, but the generalization error of SqueezeNet is larger than that of AlexNet. Hence, AlexNet achieved the best performance in our problem.

**Figure 10:**
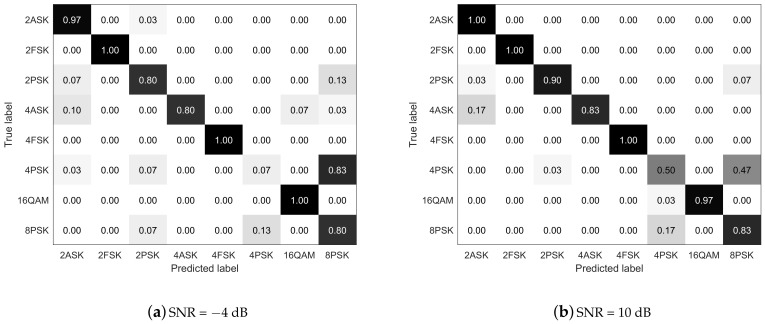
Confusion matrices of the proposed scheme in MIMO networks.

More specifically, the confusion matrices of the classification results are shown in Figure 10a,b for modulated SNRs of −4 and 10 dB, respectively. The MFSK- and QAM-modulated signals have the highest classification accuracies at both −4 and 10 dB, and the MASK-modulated signals have the second highest. The MPSK signals (especially the 4PSK signals) exhibit the worst classification performance, as shown in Figure 10a. Most of the 4PSKs are misclassified as 8PSK at SNR = −4 dB, and the performance is improved only slightly at SNR = 10 dB. This result indicates that the MIMO system structure has negative effects on the time–frequency characteristics of the MPSK signals, which is consistent with the theoretical analysis. Hence, our proposed scheme has difficulty identifying the high-order PSK signals in the MIMO system. However, the time–frequency analysis and deep-learning-based scheme have excellent performance in classifying the MFSK-, ASK-, and QAM-modulated signals, and they can obtain superior average classification accuracy for the MIMO system.

**Figure 11:**
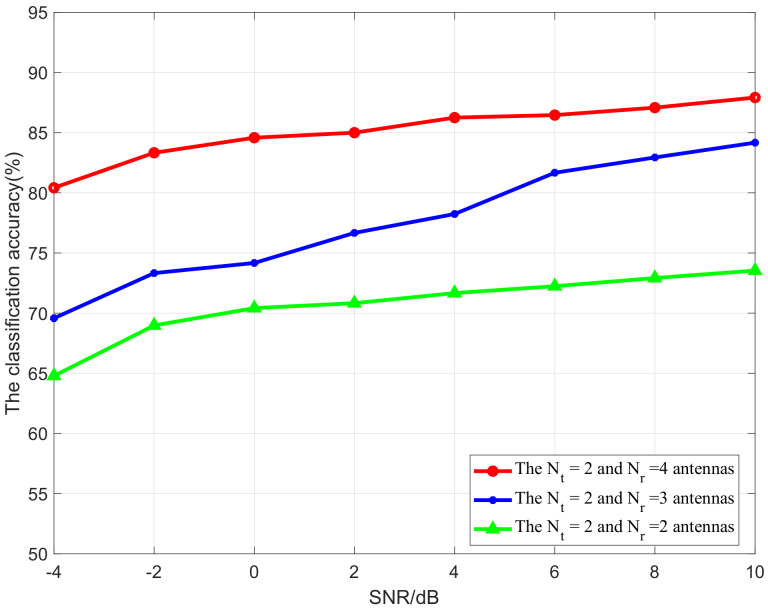
Classification accuracy versus SNR for MIMO networks with different antenna configurations.

In order to completely characterize the performance of the proposed method, we further investigate how the antenna configuration impacts the obtained performance, i.e., 2 × 2, 2 × 3, and 2 × 4 antennas over the transceiver, and the result is shown in Figure 11. One can note that, on the one hand, the classification accuracy of the proposed scheme increases as the SNR of the modulated signals increases for all different antenna configurations. On the other hand, with the increase of the number of receiving antennas, the classification accuracy is increased. These results are as expected, since the increase of the number of receiving antennas at the receiver will improve the diversity performance of the MIMO-based system; then, the decision fusion algorithm can obtain the co-operation gained by jointly deciding the correct modulation with greater probability, and thereby improving the probability of identification. However, it is important to note that, herein, for our proposed scheme, we can obtain the diversity gain for the multiple antennas receiving at the receiver and also the co-operation gain for the decision fusion scheme. Since we do not perform the estimation of the channel, it is not possible to further obtain a coherent combination gain with the multiple antennas at the receiver.

**Figure 12:**
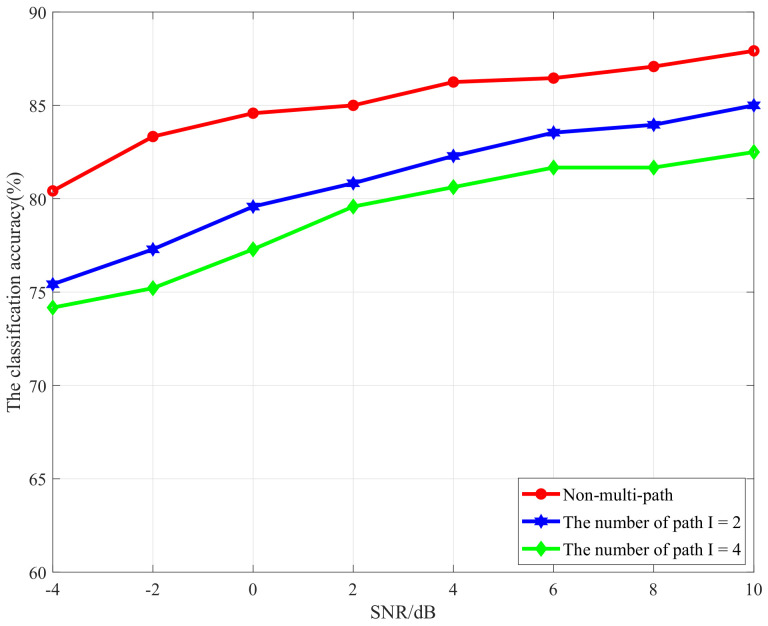
Classification accuracy versus SNR in different MIMO multi-path fading channels.

At last, the performance of the proposed approach is evaluated under more realistic multi-path fading channels for the MIMO system, and the multi-path channel model for the MIMO network is developed as follows:

(16) $$y_{j}\left(t\right)=\sum_{i=1}^{N_{t}}\sum_{p=1}^{I}h_{p}x_{i}\left(t-\tau_{p}\right),$$

where *I* denotes the number of paths for each antenna, and $y_{j}\left(t\right)$ denotes the received signal at the *j*-th antenna with the channel gain $h_{p}$ and delay $\tau_{p}$ [45]. In this, with different multi-path numbers for the MIMO channel, i.e., I=2, I=4, and non-multi-path, the classification accuracy performance is evaluated over different SNRs, and the result is shown in Figure 12. One can note that, with the increase of the number of the multiple paths, the classification accuracy is decreased. This result is consistent with the theoretical analysis, as multi-path transmission from the transmitter to receiver will cause frequency-selective fading for the signals, and the greater the number of the multiple paths is, the stronger the influence on the time–frequency properties of modulated signals will be and the more the STFT time–frequency image will be damaged. However, we can observe that, even for the scenario of path=4, the proposed scheme can still obtain 74.5% and 82.5% classification accuracy at SNR = −4 dB and SNR = 10 dB. This result indicates that our proposed scheme can obtain robust performance even for the frequency-selective channel.

#### 4.2.2. Classification Accuracy in the SISO Scenario

**Figure 13:**
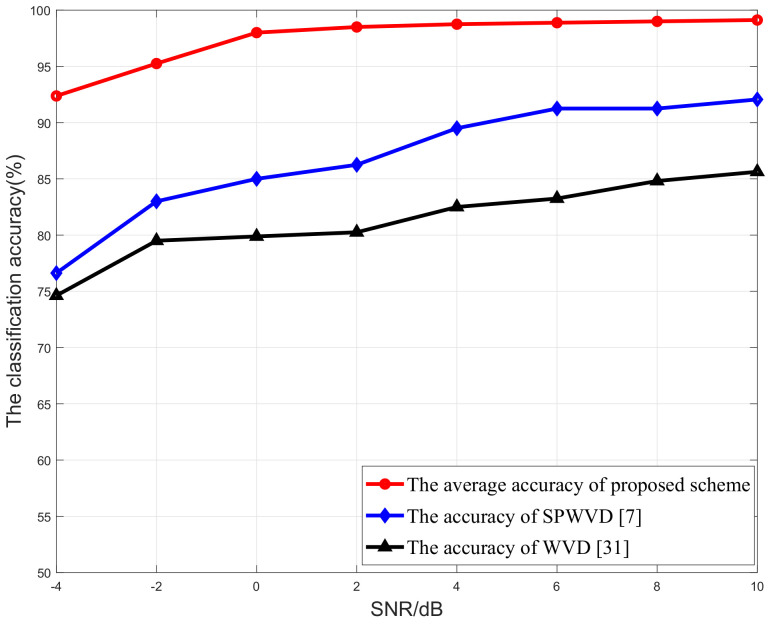
Classification accuracy versus SNR for SISO networks.

In order to better understand how different time–frequency analysis tools affect the performance of the proposed scheme, herein, both the smooth pseudo Wigner–Ville distribution (SPWVD)-based scheme [7] and the Wigner–Ville distribution (WVD)-based scheme [31] are introduced and compared with our proposed method for the SISO network, and the result is shown in Figure 13. In the simulation, the average classification accuracy of the proposed scheme is evaluated by varying the SNR of the signals from –4 to 10 dB. From this figure, we observe that as the SNRs of the signal increase, the classification accuracies of all three classification schemes are gradually improved. Moreover, our proposed scheme always has the highest average classification accuracy. Its classification accuracy is always larger than 92.37% even at SNR = −4 dB, and it has a classification accuracy of 99.12% at SNR = 10 dB. This significantly outperforms the SPWVD-based method and the WVD-based method. These results confirmed that our method has higher classification accuracy and better robustness, even in the low-SNR region. In addition, by comparing with Figure 8, we note that the average classification accuracy of the MIMO scenario is lower than that of the SISO scenario. This is due to the fact that, by using multiple antennas at the transmitter, the signals from different transmitter antennas may non-coherently combine at each receiver antenna, thus worsening the classification performance, as mentioned in Section 4.1.

## 5. Conclusions

In this paper, we resolve the problem of blind modulation classification (BMC) for MIMO systems. Specifically, the windowed STFT was used to analyze the time–frequency characteristics of the modulation signals, and the time–frequency graphs of the modulated signals were converted into RGB spectrogram images. Then, transfer learning was utilized to fine-tune AlexNet to adapt to our classification problem, and the generated RGB spectrogram images were fed into the fine-tuned CNN to extract features and train the network. Finally, the decisions of each received signal from the MIMO receivers were combined by the decision fusion module for the final decision. The STFT-based time–frequency analysis results showed that each modulation type had unique time–frequency characteristics, and that the additive noise had limited influence on the time–frequency characteristics of the modulation signals. The numerical results indicated that the proposed scheme can achieve 92.37% and 99.12% classification accuracy at the SNRs of −4 and 10 dB, respectively, in the SISO system. For the MIMO system, the proposed scheme can still achieve 80.42% and 87.78% at the SNR of −4 dB for the large and small modulation sets, respectively. This is a considerable improvement over existing studies of the BMC of MIMO systems, especially in the low-SNR region. However, many open problems still exist for BMC. As mentioned earlier, the extension of our proposed deep-learning-based approach to the MIMO OFDM system is still a challenge issue, and this is part of our future work.

## Figures and Tables

**Table 1:** Related literature on modulation classification (MC) for multiple-input multiple-output (MIMO) networks.

Literature	Scenario	Method	Features	Classes	SNR (dB)	Accuracy (%)
[20]	MIMO	FB-based	HOC, HOM ${}^{1}$	7	[−2,20]	[55.0,98.0]
[21]	MIMO	FB-based	HOC	6	[−5,15]	[56.0,98.4]
[22]	MIMO	clustering based	–	4	[−10,15]	[41.0,100.0]
[23]	MIMO, time-selective	FB-based	HOC, HOM	6	[−10,10]	[62.0,100.0]
[24]	MIMO, frequency-selective	FB-based	HOM	4	[−10,12]	[52.0,100.0]
[26,27,28,29]	MIMO-OFDM	FB-based etc.	–	–	–	–

${}^{1}$ HOC: higher-order cumulants, HOM: higher-order moments.

**Table 2:** The parameters of AlexNet.

Parameter	Number and Value
Convolution layer	5
Full connected layer	3
MiniBatchSize	10
MaxEpochs	10
Learning rate	$1.0\times 10^{-4}$

**Table 3:** The hyperparameters of the compared methods.

Name	Main Hidden Layer and Number of Layers	MiniBatchSize	MaxEpochs	Learning Rate
AlexNet	Convolution layer, 5	10	10	$1.0\times 10^{-4}$
GoogleNet	Inception, 9; Convolution layer, 2	10	6	$1.0\times 10^{-4}$
SqueezeNet	Fire, 8; Convolution layer, 2	40	20	$1.0\times 10^{-4}$

## Data Availability

Not applicable.

## Appendix A

### Appendix A.1. The Time-Domain Expression of Modulated Signals

The time-domain expression of MASK-modulated signals is described as

(A1) $$x\left(t\right)=\sum_{n}A_{m}g\left(t-nT_{s}\right)cos\left(2\pi f_{c}t+\varphi_{0}\right),$$

where $A_{m}$, $T_{s}$, $f_{c}$, and $\varphi_{0}$ represent the modulation amplitude, symbol period, carrier frequency, and initial phase, respectively. The value of $A_{m}$ depends on the symbol sequence and the modulation order *M*. In addition, g(t) is a baseband signal waveform and is usually a square-root raised cosine pulse.

Similarly, the time-domain expressions of MFSK and MPSK are defined as

(A2) $$x\left(t\right)=\sum_{n}g\left(t-nT_{s}\right)cos\left(2\pi f_{m}t+\varphi_{0}\right)$$

and

(A3) $$x\left(t\right)=\sum_{n}g\left(t-nT_{s}\right)cos\left(2\pi f_{c}t+\varphi_{m}+\varphi_{0}\right),$$

respectively.

In (A2) and (A3), $f_{m}$ and $\varphi_{m}$ are the modulation frequency and phase, respectively, and the values of these parameters depend on the symbol sequence and the modulation order *M*.

However, the QAM signal is slightly different from the MXSK (MASK, MFSK, and MPSK)-modulated signals because the QAM-modulated signal has two orthogonal carriers. Therefore, it can be represented as

(A4) $$\begin{array}{cc} x(t) & =\sum_{n}a_{n}g\left(t-nT_{s}\right)cos\left(2\pi f_{c}t+\varphi_{0}\right)\\ \; & +\sum_{n}b_{n}g\left(t-nT_{s}\right)sin\left(2\pi f_{c}t+\varphi_{0}\right), \end{array}$$

where $a_{n},b_{n}\in \left[2m-1-\sqrt{M}\right]$, $m=1,2,\ldots ,\sqrt{M}$, and the two carriers are individually modulated by $a_{n}$ and $b_{n}$ [10].

### Appendix A.2. Theory of the STFT

Consider a signal s(τ) and a real, even window w(τ), whose Fourier transforms (FTs) are S(f) and W(f), respectively. To obtain a localized spectrum of s(τ) at time τ=t, the signal is multiplied by the window w(τ) centered at time τ=t, which results in

(A5) $$s_{w}\left(t,\tau \right)=s\left(\tau \right)w\left(\tau -t\right).$$

Next, the FT is taken at time τ, obtaining

(A6) $$F_{s}^{w}\left(t,f\right)=\underset{\tau \to f}{F}s\left(\tau \right)w\left(\tau -t\right),$$

where $F_{s}^{w}\left(t,f\right)$ is the STFT [31]. It is known that the time and frequency resolutions of the STFT depend on the nature and length of the window w(τ), and we cannot achieve high resolutions in time and frequency simultaneously. However, a tradeoff between time and frequency resolution can be achieved by adjusting the length and overlapping length of the window to obtain better time–frequency analysis performance.

## References

[1] Gupta R., Majhi S., Dobre O.A. (2019). Design and Implementation of a Tree-Based Blind Modulation Classification Algorithm for Multiple-Antenna Systems. IEEE Trans. Instrum. Meas..

[2] Liao K., Tao G., Zhong Y., Zhang Y., Zhang Z. (2019). Sequential Convolutional Recurrent Neural Networks for Fast Automatic Modulation Classification. arXiv.

[3] Eldemerdash Y.A., Dobre O.A., Öner M. (2016). Signal Identification for Multiple-Antenna Wireless Systems: Achievements and Challenges. IEEE Commun. Surv. Tutor..

[4] Dobre O.A., Abdi A., Bar-Ness Y., Su W. (2007). Survey of automatic modulation classification techniques: Classical approaches and new trends. IET Commun..

[5] Xu J.L., Su W., Zhou M. Likelihood function-based modulation classification in bandwidth-constrained sensor networks. Proceedings of the 2010 International Conference on Networking, Sensing and Control (ICNSC).

[6] Abdelbar M., Tranter W.H., Bose T. (2018). Cooperative Cumulants-Based Modulation Classification in Distributed Networks. IEEE Trans. Cogn. Commun. Netw..

[7] Zhang Z., Wang C., Gan C., Sun S., Wang M. (2019). Automatic Modulation Classification Using Convolutional Neural Network with Features Fusion of SPWVD and BJD. IEEE Trans. Signal Inf. Process. Netw..

[8] Harjani R., Cabric D., Markovic D., Sadler B.M., Palani R.K., Saha A., Shin H., Rebeiz E., Basir-Kazeruni S., Yuan F. (2015). Wideband blind signal classification on a battery budget. IEEE Commun. Mag..

[9] Han L., Gao F., Li Z., Dobre O.A. (2017). Low Complexity Automatic Modulation Classification Based on Order-Statistics. IEEE Trans. Wirel. Commun..

[10] Wu Z., Zhou S., Yin Z., Ma B., Yang Z. (2017). Robust Automatic Modulation Classification Under Varying Noise Conditions. IEEE Access.

[11] Shah S.I.H., Alam S., Ghauri S.A., Hussain A., Ahmed Ansari F. (2019). A Novel Hybrid Cuckoo Search- Extreme Learning Machine Approach for Modulation Classification. IEEE Access.

[12] Li W., Dou Z., Lin Y., Shi C. (2019). Wavelet transform based modulation classification for 5G and UAV communication in multipath fading channel. Phys. Commun..

[13] O’Shea T., Hoydis J. (2017). An Introduction to Deep Learning for the Physical Layer. IEEE Trans. Cogn. Commun. Netw..

[14] Ramjee S., Ju S., Yang D., Liu X., Gamal A.E., Eldar Y.C. (2019). Fast Deep Learning for Automatic Modulation Classification. arXiv.

[15] Rajendran S., Meert W., Giustiniano D., Lenders V., Pollin S. (2018). Deep Learning Models for Wireless Signal Classification with Distributed Low-Cost Spectrum Sensors. IEEE Trans. Cogn. Commun. Netw..

[16] Nie J., Zhang Y., He Z., Chen S., Gong S., Zhang W. (2019). Deep Hierarchical Network for Automatic Modulation Classification. IEEE Access.

[17] Meng F., Chen P., Wu L., Wang X. (2018). Automatic Modulation Classification: A Deep Learning Enabled Approach. IEEE Trans. Veh. Technol..

[18] Ma J., Lin S., Gao H., Qiu T. Automatic Modulation Classification Under Non-Gaussian Noise: A Deep Residual Learning Approach. Proceedings of the 2019 IEEE International Conference on Communications (ICC).

[19] O’Shea T.J., Corgan J., Clancy T.C., Jayne C., Iliadis L. (2016). Convolutional Radio Modulation Recognition Networks. Engineering Applications of Neural Networks.

[20] Hassan K., Dayoub I., Hamouda W., Nzeza C.N., Berbineau M. (2012). Blind Digital Modulation Identification for Spatially-Correlated MIMO Systems. IEEE Trans. Wirel. Commun..

[21] Kharbech S., Dayoub I., Zwingelstein-Colin M., Simon E.P. (2016). On classifiers for blind feature-based automatic modulation classification over multiple-input-multiple-output channels. IET Commun..

[22] Tian J., Pei Y., Huang Y., Liang Y. A Machine Learning Approach to Blind Modulation Classification for MIMO Systems. Proceedings of the 2018 IEEE International Conference on Communications (ICC).

[23] Kharbech S., Dayoub I., Zwingelstein-Colin M., Simon E.P. (2018). Blind Digital Modulation Identification for MIMO Systems in Railway Environments With High-Speed Channels and Impulsive Noise. IEEE Trans. Veh. Technol..

[24] Marey M., Dobre O.A. (2016). Blind Modulation Classification Algorithm for Single and Multiple-Antenna Systems Over Frequency-Selective Channels. IEEE Signal Process. Lett..

[25] Marey M., Dobre O.A. (2015). Blind Modulation Classification for Alamouti STBC System With Transmission Impairments. IEEE Wirel. Commun. Lett..

[26] Liu Y., Simeone O., Haimovich A.M., Su W. (2017). Modulation Classification for MIMO-OFDM Signals via Approximate Bayesian Inference. IEEE Trans. Veh. Technol..

[27] Liu Y., Simeone O., Haimovich A.M., Su W. Modulation classification for MIMO-OFDM signals via Gibbs sampling. Proceedings of the 2015 49th Annual Conference on Information Sciences and Systems (CISS).

[28] Agirman-Tosun H., Liu Y., Haimovich A.M., Simeone O., Su W., Dabin J., Kanterakis E. Modulation classification of MIMO-OFDM signals by Independent Component Analysis and Support Vector Machines. Proceedings of the 2011 Conference Record of the Forty Fifth Asilomar Conference on Signals, Systems and Computers (ASILOMAR).

[29] Haring L., Kisters C. Signaling-Assisted MAP-Based Modulation Classification in Adaptive MIMO OFDM Systems. Proceedings of the 2014 IEEE 80th Vehicular Technology Conference (VTC2014-Fall).

[30] Gao M., Li Y., Dobre O.A., Al-Dhahir N. (2019). Joint Blind Identification of the Number of Transmit Antennas and MIMO Schemes Using Gerschgorin Radii and FNN. IEEE Trans. Wirel. Commun..

[31] Boashash B. (2016). Time-Frequency Signal Analysis and Processing.

[32] Ozer I., Ozer Z., Findik O. (2018). Noise robust sound event classification with convolutional neural network. Neurocomputing.

[33] Öner M. (2018). On The Classification of Binary Space Shift Keying Modulation. IEEE Commun. Lett..

[34] Câmara T.V.R.O., Lima A.D.L., Lima B.M.M., Fontes A.I.R., Martins A.D.M., Silveira L.F.Q. (2019). Automatic Modulation Classification Architectures Based on Cyclostationary Features in Impulsive Environments. IEEE Access.

[35] Proakis J.G., Salehi M. (2001). Digital Communications.

[36] Kim B., Kong S., Kim S. (2015). Low Computational Enhancement of STFT-Based Parameter Estimation. IEEE J. Sel. Top. Signal Process..

[37] Mitra S.K., Kuo Y. (2006). Digital Signal Processing: A Computer-Based Approach.

[38] Mathworks Jet Color Chart Array. https://ww2.mathworks.cn/help/matlab/ref/jet.html.

[39] Krizhevsky A., Sutskever I., Hinton G. (2012). ImageNet Classification with Deep Convolutional Neural Networks. Adv. Neural Inf. Process. Syst..

[40] Rezaee M., Mahdianpari M., Zhang Y., Salehi B. (2018). Deep Convolutional Neural Network for Complex Wetland Classification Using Optical Remote Sensing Imagery. IEEE J. Sel. Top. Appl. Earth Obs. Remote Sens..

[41] Pan S.J., Yang Q. (2010). A Survey on Transfer Learning. IEEE Trans. Knowl. Data Eng..

[42] Atapattu S., Tellambura C., Hai J. (2014). Energy Detection for Spectrum Sensing in Cognitive Radio.

[43] Iandola F.N., Moskewicz M.W., Ashraf K., Han S., Dally W.J., Keutzer K. (2016). SqueezeNet: AlexNet-level accuracy with 50x fewer parameters and <1 MB model size. arXiv.

[44] Szegedy C., Liu W., Jia Y., Sermanet P., Reed S., Anguelov D., Erhan D., Vanhoucke V., Rabinovich A. Going deeper with convolutions. Proceedings of the 2015 IEEE Conference on Computer Vision and Pattern Recognition (CVPR).

[45] Cho Y.S. (2010). MIMO-OFDM Wireless Communications with Matlab.

